# Selected Inflammatory and Metabolic Markers in Psoriatic Patients Treated with Goeckerman Therapy

**DOI:** 10.1155/2015/979526

**Published:** 2015-06-08

**Authors:** Katerina Kondelkova, Lenka Borska, Ctirad Andrys, Jan Krejsek, Kvetoslava Hamakova, Simona Rendarova, Vit Rehacek, Jan Kremlacek, Zdenek Fiala

**Affiliations:** ^1^Institute of Clinical Immunology and Allergology, University Hospital Hradec Kralove, Faculty of Medicine in Hradec Kralove, Charles University in Prague, Sokolska 581, 50005 Hradec Kralove, Czech Republic; ^2^Institute of Pathological Physiology, Faculty of Medicine in Hradec Kralove, Charles University in Prague, Sokolska 581, 50005 Hradec Kralove, Czech Republic; ^3^Department of Dermatology and Venereal Diseases, University Hospital Hradec Kralove, Sokolska 581, 50005 Hradec Kralove, Czech Republic; ^4^Department of Transfusion Medicine, University Hospital Hradec Kralove, Sokolska 581, 50005 Hradec Kralove, Czech Republic; ^5^Institute of Hygiene and Preventive Medicine, Faculty of Medicine in Hradec Kralove, Charles University in Prague, Sokolska 581, 50005 Hradec Kralove, Czech Republic

## Abstract

Psoriasis is associated with metabolic activity of adipose tissue which produces pro- and anti-inflammatory adipokines. Goeckerman therapy (GT) represents an effective treatment of psoriasis. This study evaluated variation of selected inflammatory and metabolic markers during GT and the relationships between the markers, severity of the disease (PASI score), body mass, and the basic characteristics of the therapy. The study was conducted on a group of patients (*n* = 32) and on a control group (*n* = 24). Before GT, we found significantly elevated levels of proinflammatory CRP (*p* < 0.001) and leptin (*p* < 0.05) in psoriatic patients (compared to the controls). The therapy significantly decreased the levels of CRP and adiponectin. We found positive correlations between CRP and total duration of GT (*p* < 0.05) and CRP and the time of UV exposure (*p* < 0.01) and negative correlations between adiponectin and the total duration of GT (*p* < 0.05) and adiponectin and the application of CCT ointment (*p* < 0.001). From our results, we can conclude that GT causes partial reduction of both proinflammatory and anti-inflammatory markers. However, the levels of proinflammatory CRP and leptin remained significantly higher in the patients than in the control group.

## 1. Introduction

Psoriasis is a systemic, predominantly T cell-driven inflammatory skin disorder associated with serious comorbidities such as obesity, diabetes, cardiovascular diseases, and metabolic syndrome [1, 2]. Psoriasis affects approximately 2.5% of the world population [3, 4]. The prevalence of the metabolic syndrome is increased in psoriatic patients and occurs in 40% or even 65% of them [4]. It was substantiated that overweight is an independent risk factor for developing psoriasis and that obesity may increase the risk more than twice [4].

It seems that the processes of initiation and development of psoriasis and overweight/obesity are associated with various forms of chronic inflammation. Moreover, Ryan and Kirby summarized that suppression of systemic inflammation in psoriasis could also reduce metabolic inflammation [5].

Adipose tissue is mainly composed of adipocytes which are able to secrete a wide range of protein-cytokines called adipokines. Adipokines are involved in the regulation of metabolism, insulin sensitivity, and inflammation [6]. Serum concentrations of different adipokines are associated with obesity and metabolic and cardiovascular diseases. It is hypothesized nowadays that adipokines serum concentration may serve as predictors of mortality in obesity related diseases [7]. Besides these functions, adipokines also interact with immune cells, therefore contributing to the inflammatory network.

Adipose tissue produces proinflammatory and anti-inflammatory adipokines to modulate inflammatory responses and to regulate feeding behavior [8]. Adiponectin and leptin are members of the adipokines family. Leptin has proinflammatory effects. It activates monocytes and macrophages to produce proinflammatory IL-6, TNF-*α*, and IL-12. Leptin also enhances the production of proinflammatory Th1 cytokines and suppresses the production of anti-inflammatory Th2 cytokines at the same time. Adiponectin is highly expressed by adipocytes with potent anti-inflammatory properties. Adiponectin inhibits TNF-*α*, IL-6, and INF-*γ* production, phagocytic activity of macrophages, and expression of monocyte cell adhesion molecules and stimulates the production of anti-inflammatory IL-10. It also modulates T cell activation and inflammatory function of NK cells [8, 9].

Metabolic inflammation also increases the level of nonspecific inflammatory biomarkers, such as C-reactive protein (CRP). The level of inflammatory response CRP strongly correlates with the degree of obesity [6, 10].

Goeckerman therapy (GT) represents an effective treatment of moderate to severe psoriasis [11–13]. At present, this older therapy is becoming increasingly important, especially in the context of increasing number of patients with biological forms of therapy and related cases of resistance to biologic treatment.

GT is based on daily dermal application of pharmaceutical grade crude coal tar (CCT) ointment with high portion of polycyclic aromatic hydrocarbons (PAHs) and subsequent whole body exposure to UV radiation (A, B) [12, 14].

However, there are some safety concerns addressing this therapy. Many PAHs are recognized as carcinogens with tumor-initiating and/or tumor-promoting properties [15]. It is likely that UV radiation used in GT pronounces the risk of mutagenicity, carcinogenicity, and immunotoxicity [16, 17]. The increased risk of nonmelanoma skin cancer in patients treated with crude coal tar (with or without exposure to UV radiation) was found by several studies [18, 19], whereas other studies did not reveal the risk [20–22].

Reliable biomarkers are essential for determining the severity of disease and related health hazards and for evaluation of efficacy of the therapy. To our best knowledge, no work addressing the changes in levels of adipokines during the Goeckerman therapy of psoriasis has been carried out so far. The presented study evaluated (1) the variation of selected inflammatory and metabolic markers during Goeckerman therapy of psoriasis and (2) the relationships between the selected markers, severity of the disease, body mass, and basic characteristics of the therapy.

## 2. Materials and Methods

### 2.1. Observed Group

Two groups were investigated in this study. The first group consisted of patients at active stage of chronic stable plaque psoriasis, hospitalized at Clinic of Dermal and Venereal Diseases, University Hospital Hradec Kralove (Czech Republic) and treated by GT. The clinical data were being collected for two years (from January 2012 to November 2013). The group consisted of 13 women and 19 men (average age of 57 years, age range 21–75 years, 14 smokers and 18 nonsmokers). Recent exposure history of the patients was regularly checked using a questionnaire (exposure to UV radiation and PAHs, including smoking). Those patients who had had significant occupational or nonoccupational exposure to PAHs and/or artificial UVR were excluded from the monitored group. Subjects with insulin resistance, diabetes, cardiovascular diseases, infections, or other inflammatory diseases (diagnosed either before or during the treatment) were excluded from the study. Our patients were not treated by any drugs influencing inflammatory reaction. No further medications regarding psoriasis were administered to our patients at least 3 months before starting treatment. No patients suffering from psoriatic arthritis were enrolled to our study.

The second (control) group consisted of healthy blood donors (10 women, 14 men; average age of 43 years, age range 22–65 years, 9 smokers and 15 nonsmokers).

The rate of overweight/obesity was evaluated using BMI (Body Mass Index). BMI was calculated as the ratio of weight and height squared (kg/m^2^).

The study was approved by the Ethics Committee of the University Hospital in Hradec Kralove, Czech Republic. Informed written consent was obtained from each patient.

### 2.2. Goeckerman Therapy

During the therapy, the dermatological ointment containing 5% of CCT was administered daily (overnight) on psoriatic lesions. According to the extent of lesions, 16–58% of the total body surface was covered by CCT ointment. Each morning the residues of CCT ointment were removed from the body surface (using oil bath) and the patients were whole-body irradiated by UVR. UVR exposures were dosed according to the skin condition and temporarily omitted in cases of adverse reactions. For this reason, the total duration of GT (in days) is not equivalent to the frequency of UVR exposure. Irradiation time was individual according to the disease status (1–15 min/day). The density of UV radiation used was 248.55 *μ*W/cm^2^ of UV-B and 130.1 *μ*W/cm^2^ of UV-A (controlled by Sola-Scope 2000 spectrometer; Solatell, UK). Duration of the treatment was modified according to the treatment effectiveness and the therapy was ceased when significant improvement in clinical status was achieved (average duration of 13 days; range of 10–19 days).

### 2.3. Selected PAHs in CCT

Determination of selected PAHs in the CCT has been described previously [23].

### 2.4. C-Reactive Protein, Leptin, and Adiponectin

Samples of blood were collected from the cubital vein before the first treatment and immediately after the last medical procedure (BD Vacutainer sampling tubes). Blood serum was isolated by centrifugation and stored at –70°C until analysis. Repeated thawing and freezing were avoided.

The level of C-reactive protein (CRP) was assessed by immunonephelometry on IMMAGE 800 (Beckman, USA). The level of CRP was expressed in milligrams (mg) per liter of serum with detection limit of 1.0 mg per liter.

The levels of leptin and adiponectin were detected by sandwich enzyme-linked immunosorbent assay kits (ELISA) for human leptin and human total adiponectin/Acrp30 (R&D Systems, USA). The assays were run according to the instruction provided by the manufacturer. Plates were read at 450 nm with the microplate reader Multiskan (Thermo Fisher Scientific, USA).

The concentration of leptin was expressed in micrograms (*μ*g) per liter of serum with detection limit of 0.0078 *μ*g of species per liter of serum. The level of adiponectin was expressed in milligrams (mg) per liter of serum with detection limit of 0.000246 mg per liter.

### 2.5. PASI Score

The effectiveness of the therapy was calculated from basic characteristics of actual disease status (erythema, desquamation, and skin infiltration) and expressed as the PASI score (Psoriasis Area and Severity Index) [24].

### 2.6. Statistical Analysis

Obtained data were analyzed by using MATLAB rel. 2014a software (Mathworks, Inc., Massachusetts, USA). Because the Lilliefors test of normality had rejected the hypothesis of normal distribution, the data before and after GT treatment were analyzed by the Wilcoxon signed rank test and for intergroup comparison the Wilcoxon rank sum test was used. To exclude the confounding effect of different age, smoking habits, and gender presentation in patients and controls, Wilcoxon rank sum test and chi-squared tests were carried out. The possible associations between monitored biomarkers and important factors of exposure were evaluated by using of Spearman Rank Order test. The findings were considered as significant when the probability level (*p*) was below 0.05.

## 3. Results

The determination of selected PAHs in the CCT has been described in previous work [23]. Briefly, for the determination, we used a pooled sample of CCT and analyses were done for 16 selected PAHs (according to the US Environmental Protection Agency). The total content of 16 selected PAHs was 9.546 mg/g of CCT. In the amount of 100 g of 5% CCT ointment used there was approximately 47.7 mg of 16 selected PAHs.

**Figure 1:**
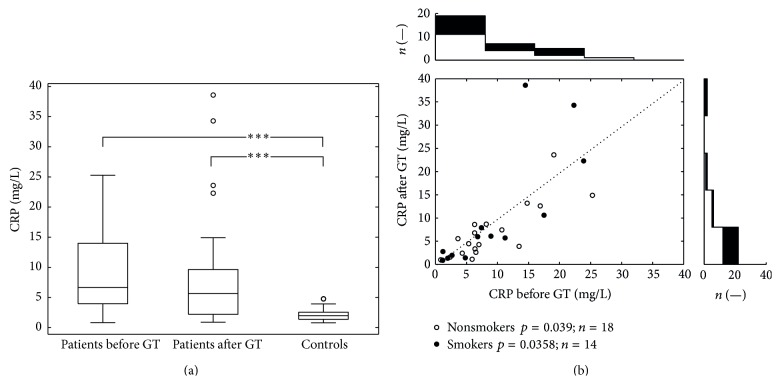
The variation in CRP values. (a) depicts intergroup comparisons of serum levels of CRP using Wilcoxon rank sum test (patients before and after GT versus controls). Patients showed before and after GT significantly higher CRP levels than controls (*p* < 0.001). (b) (scatter plot) depicts serum CRP levels before and after GT in patients. Pairwise comparison (Wilcoxon signed rank test) indicated a decrease of the CRT level (*p* < 0.05). For smokers (black dots) and nonsmokers (open circles) statistics see the plot legend. All together 32 dots represent 64 measurements; each dot belongs to one patient. The top histogram shows data distribution before the treatment; the right side histogram corresponds to the posttreatment values distribution.

Figure 1 presents the differences in the levels of CRP. After GT, we found significantly decreased serum levels of CRP in the group of patients (*p* < 0.05). When compared to the group of healthy controls (median 1.97, interquartile range 1.37–2.55), serum levels of CRP in the group of patients were significantly higher both before (median 6.67, interquartile range 3.97–14.0; *p* < 0.001) and after (median 5.65, interquartile range 2.20–9.63; *p* < 0.001) the therapy. The white points (zones) in the lower part of Figure 1 represent individual data of nonsmokers (significantly lower after GT, *p* = 0.039), and the black points (zones) represent individual data of smokers (not significant change of values after GT, *p* = 0.358).

**Figure 2:**
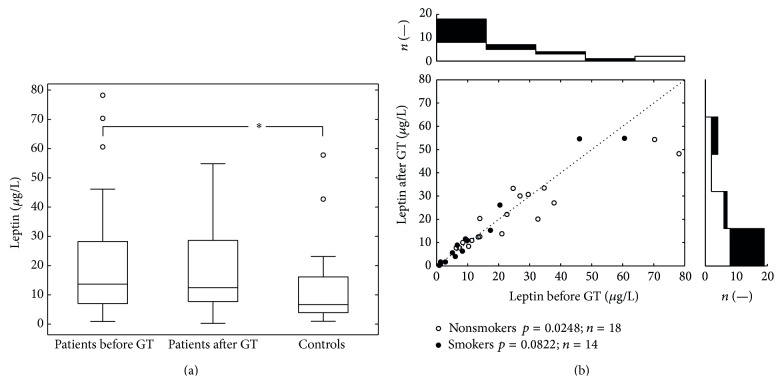
The variation in leptin values. (a) depicts intergroup comparisons of serum levels of leptin using Wilcoxon rank sum test (patients before and after GT versus controls). Patients before GT had significantly higher level of the leptin than controls (*p* < 0.05). (b) (scatter plot) depicts serum leptin levels before and after GT in patients. Pairwise comparison (Wilcoxon signed rank test) did not show any statistically significant difference. For smokers (black dots) and nonsmokers (open circles) statistics see the plot legend. All together 32 dots represent 64 measurements; each dot belongs to one patient. The top histogram shows data distribution before the treatment; the right side histogram corresponds to the posttreatment values distribution.

Similar to the level of CRP, the leptin level in the group of patients (before the treatment) was found to be significantly higher (median 13.67, interquartile range 7.05–28.25) than leptin level in the control group (median 6.61, interquartile range 3.96–16.15; *p* < 0.05). After the therapy (median 12.47, interquartile range 7.73–28.6) the leptin level decreased only insignificantly; however, this decline was sufficient to abolish the difference between the patient (after therapy) and the control group (Figure 2). The white points (zones) in the lower part of Figure 2 represent individual data of nonsmokers and the black points (zones) represent individual data of smokers. Statistically significant differences in leptin levels (between the levels before and after GT) were not observed even after further stratification of the group of patients to smokers and nonsmokers (*p* = 0.822 and *p* = 0.248).

**Figure 3:**
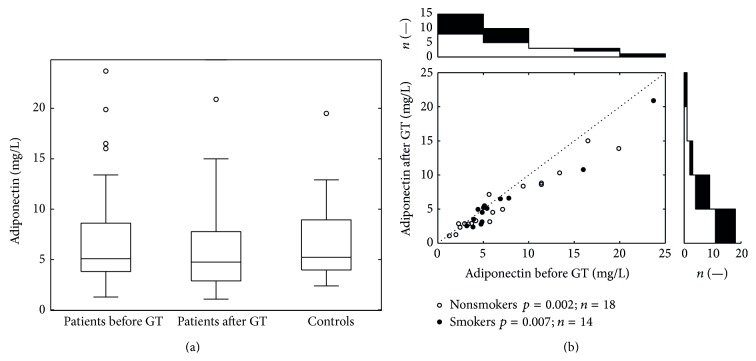
The variation in adiponectin values. (a) depicts intergroup comparisons of serum levels of adiponectin using Wilcoxon rank sum test (patients before and after GT versus controls). The test did not show any intergroup differences. (b) (scatter plot) depicts serum adiponectin levels before and after GT in patients. Pairwise comparison of patients (before versus after GT) by Wilcoxon signed rank test showed significantly higher levels of adiponectin before GT for the whole group (*p* < 0.001). For smokers (black dots) and nonsmokers (open circles) statistics see the plot legend. All together 32 dots represent 64 measurements; each dot belongs to one patient. The top histogram shows data distribution before the treatment; the right side histogram corresponds to the after treatment values distribution.

Serum levels of adiponectin in psoriatic patients both before (median 5.07, interquartile range 3.79–8.59) and after (median 4.74, interquartile range 2.87–7.75) treatment were lower than the level of adiponectin in the control group (median 5.22, interquartile range 3.96–8.93). However, the differences did not reach statistical significance. On the other hand, the decrease in adiponectin levels after the therapy was significant (*p* < 0.001) (Figure 3). The white points (zones) in the lower part of Figure 3 represent individual data of nonsmokers and the black points (zones) represent individual data of smokers. When we stratified the patients into subgroups of smokers and nonsmokers, we found a significant decrease in levels of adiponectin (*p* = 0.007 and *p* = 0.002) after the therapy in both subgroups. We can therefore assume that smoking had probably no significant effect on adiponectin level.

The degree of severity of psoriasis (expressed as PASI score) was significantly decreased after the therapy (*p* < 0.001). Median, lower, and upper quartile of values of PASI score were 17.1 (14.5–20.6) before the therapy and 6.0 (4.3–7.9) after the therapy. We did not find significant relationships between the levels of observed inflammatory and metabolic biomarkers, PASI score (both before and after therapy), and age of participants. On the other hand we found significant relationships between basic characteristics of GT (length/range) and severity/range of psoriasis (PASI score). Related values of correlation coefficient were *r* = 0.35 (duration of GT versus PASI score; *p* < 0.05), *r* = 0.37 (time of UV exposure versus PASI score; *p* < 0.05), and *r* = 0.62 (range/area of daily application of CCT versus PASI score; *p* < 0.001).

Table 1 summarizes relationships among biomarkers (values observed after the treatment) and basic characteristics of the therapy. The data show significant (positive) relationships between the level of CRP, total duration of GT (*p* < 0.05), and time of UV exposure (*p* < 0.01). Significant (negative) relationships were identified between the level of adiponectin, total duration of GT (*p* < 0.05), and application of CCT ointment (*p* < 0.001). We did not find any mutual relationships between CRP, leptin, and adiponectin (data after the treatment).

The BMI level in the group of patients (median/lower-upper quartile: 28.4/24.0–32.6) did not differ significantly from the control group (median/lower-upper quartile: 26.7/24.5–28.3). Before the therapy, the relationships between BMI and biomarkers were significant for leptin (*r* = 0.59; *p* < 0.001) and adiponectin (*r* = −0.72; *p* < 0.001), and, after the therapy, they were significant for CRP (*r* = 0.40; *p* < 0.05), leptin (*r* = 0.58; *p* < 0.001), and adiponectin (*r* = −0.65; *p* < 0.001).

**Figure 4:**
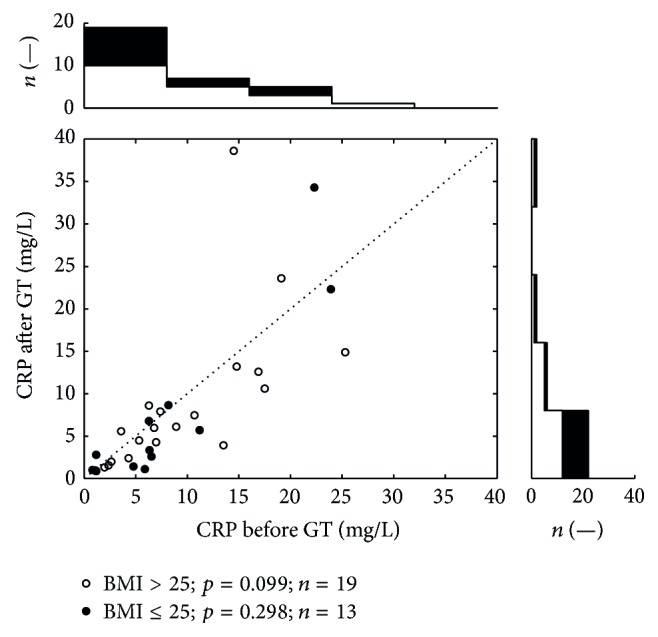
The variation in CRP values, BMI separation. The scatter plot depicts serum CRP levels before and after GT in a similar way as in Figure 1. This plot divides the group of patients by BMI value. Patients with BMI over 25 are plotted as open circles and patients with BMI below or equal to 25 are represented as black dots. All together 32 dots represent 64 measurements; each dot belongs to one patient. The top histogram shows data distribution before the treatment; the right side histogram corresponds to the after treatment values distribution.

To evaluate the effect of BMI on the level of CRP (Figure 4), we divided the patients into two subgroups: BMI ≤ 25 (*n* = 19), and BMI > 25 (*n* = 13). We found (median and lower-upper quartile) 7.40 mg/L (4.58–14.73) for BMI > 25 and 6.31 mg/L (1.19–8.93) for BMI ≤ 25 before the therapy. We found 6.09 mg/L (4.01–12.10) for BMI > 25 and 2.78 mg/L (1.10–7.26) for BMI ≤ 25 after the therapy. It is apparent that there are differences in the decrease in CRP levels between individuals with different BMI, but these differences did not reach the level of statistical significance (Figure 4).

## 4. Discussion

As resulted from the analysis of selected PAHs in CCT used [23], one PAH (from the whole group of 16 PAHs) was ranked into the group of proven carcinogens, another one PAH in the group of probable carcinogens and six PAHs was ranked into the group of possible carcinogens. Remaining PAHs, despite being registered by IARC, are not classifiable as to its carcinogenicity to humans [15].

It was mentioned before that the processes of initiation and subsequent development of psoriasis and overweight/obesity are associated with chronic inflammation. In this context, the already published data support the assumption that cytokines produced by adipocytes, leptin, and adiponectin could be involved in the pathogenesis of psoriasis. Oh et al. found significantly higher serum leptin concentrations in patients with psoriasis (compared to healthy controls), while serum adiponectin level was significantly decreased. The leptin concentration in the study showed no relationships with BMI or PASI score; however, concentration of adiponectin showed negative relationships with BMI and PASI score in patients with psoriasis. This suggests that there are probably multiple independent factors involved in regulation of serum leptin and adiponectin levels [25].

Rajappa et al. observed high levels of proinflammatory adipokines (leptin, resistin, and IL-6) and lower levels of anti-inflammatory adipokines (including adiponectin) in patients with psoriasis [26]. Similar results were published also by other authors. Zhu et al. found significantly higher leptin level in patients with psoriasis compared to controls [27]. Li et al. reported significantly lower level of adiponectin and insignificantly higher level of leptin in psoriatic patients [28]. Coimbra et al. described significantly higher levels of BMI, leptin, and CRP and significantly lower level of adiponectin in psoriatic patients [29].

We observed similar trends in the levels of monitored biomarkers in our study as well. When we compared the patients with psoriasis with the controls (before the therapy), we found significantly higher levels of proinflammatory markers (CRP and leptin) and insignificantly lower level of adiponectin. Therefore, we could proclaim that elevated levels of proinflammatory CRP and leptin and decreased levels of anti-inflammatory adiponectin reflect an ongoing inflammatory process in patients with psoriasis. However, it must be taken into account that the levels of leptin and adiponectin are strongly influenced by the amount of adipose tissue in an individual patient [6].

Results concerning the levels of adipokines in psoriatic patients are, however, not consistent. Özdemir et al. presented that although the serum leptin and adiponectin levels in patients with psoriasis (before treatment) were higher when compared with the control group, the differences were not significant [30]. Also, in the study by Zhu et al., the adiponectin levels were not significantly different in patients with psoriasis compared to controls. Moreover, the authors suggested that the levels of adiponectin may not be associated with psoriasis and that the relationship between psoriasis and adiponectin needs to be clarified [31]. Likewise in the study of Corbetta et al. where the adiponectin levels in psoriatic patients did not differ from those observed in healthy control subjects [32].

Evaluation of changes in adipokines levels following different types of psoriasis treatments is still controversial. Coimbra et al. demonstrated that NBUVB therapy did not induce significant changes in the levels of leptin and adiponectin [29]. Similarly, Corbetta et al. did not observe any changes in adiponectin levels after retinoid therapy [32] and Kawashima et al. did not reveal significant changes in the levels of leptin after the phototherapy [33].

On the other hand, increase of adiponectin concentrations and reduction of CRP level were reported after PUVA therapy [29]. Özdemir et al. found increased levels of adiponectin in patients with psoriasis after cyclosporin therapy [30]. Rajappa et al. reported that the levels of inflammatory adipokines in patients treated by methotrexate remained higher compared to the controls and thus confirmed persistent inflammation [26].

We showed that Goeckerman therapy induces immunomodulatory, immunosuppressive, and anti-inflammatory effects in our previous studies [10, 12, 34]. In accordance with these results and results of other authors we observed the decrease of proinflammatory CRP and leptin after the therapy which was significant in the case of CRP. It must be stressed out that even though the levels of CRP and leptin were decreased after the therapy; they still remained elevated compared to the control group. This suggests persistent inflammation in patients with psoriasis in remission as reported in psoriasis patients treated with methotrexate [26]. Despite the effectiveness of treatment, psoriasis is still an incurable disease. Goeckerman therapy is able to induce long-term remission in the majority of patients; however, both the immune system and the skin of patients with psoriasis also show abnormalities in the period without clinical signs. This fact may be one of the reasons for the relapse of psoriasis.

Contrary to some reports [29, 30], our results showed that the levels of adiponectin decreased after GT. However, it must be emphasized that in both studies a completely different type of therapy, comprising PUVA and cyclosporine administration, was used [29, 30]. We can assume that the observed decrease in our study could have occurred due to the toxic effects of PAHs [35].

PAHs have strong lipophilic properties and are transported into all tissues of the human body containing fat. High-molecular-weight PAHs, benzo[a]pyrene, impair adipose tissue lipolysis and lead to increased weight gain and increased fat mass and could have an impact on metabolic disorders, such as obesity [36–38]. Ortiz et al. presented that early life exposure to tobacco smoke (containing PAHs) and air pollution (contaminated by PAHs) have been linked to increased risk of obesity and metabolic syndrome [35, 39]. In addition, it has been recently discovered that PAHs may play a central role in carcinogenesis not only by inducing the cancer via their mutagenic properties, but also by enhanced bioaccumulation capacity of the adipose tissue [36–38].

We found the significant negative relationship between BMI and the level of adiponectin after the therapy. We found significant (positive) relationships between the level of CRP, total duration of GT and time of UV exposure. These results were expected because more extensive inflammation requires longer treatment. However, it is interesting that these correlations were not found in the case of proinflammatory marker, leptin. In the case of adiponectin, we found significant (negative) relationships with total duration of GT and with application of CCT ointment. This phenomenon could be explained by the fact that the extent and intensity of inflammation (psoriasis) is inversely proportional to the level of anti-inflammatory factors [28].

## 5. Conclusions

In conclusion, before the treatment we observed significantly elevated levels of proinflammatory CRP and leptin in psoriatic patients, compared to the controls. The therapy significantly decreased the levels of CRP and adiponectin; however, the level of CRP remained significantly higher when compared to the controls.

We found positive correlations between CRP, the total duration of GT, and the time of UV exposure and negative correlations between adiponectin, the total duration of GT, and the application of CCT ointment.

We can conclude that GT causes a partial reduction of both proinflammatory and anti-inflammatory markers.

## Figures and Tables

**Table 1:** Relationships among biomarkers and basic characteristics of the therapy.

Biomarkers^*∗*^	Influencing factors		
	Total duration of GT (days)	Time of UV exposure (days)	Application of crude coal tar ointment (% of body surface)
CRP (mg/L)	*r* = 0.36 (*p* < 0.05)	*r* = 0.49 (*p* < 0.01)	*r* = 0.26 (NS)
Leptin (*µ*g/L)	*r* = 0.30 (NS)	*r* = 0.17 (NS)	*r* = 0.32 (NS)
Adiponectin (mg/L)	*r* = −0.40 (*p* < 0.05)	*r* = −0.25 (NS)	*r* = −0.70 (*p* < 0.001)

^*∗*^Values observed after the therapy.

*r*: value of Spearman Rank Order Correlation.
